# Identification of Protein Kinase Inhibitors with a Selective Negative Effect on the Viability of Epstein-Barr Virus Infected B Cell Lines

**DOI:** 10.1371/journal.pone.0095688

**Published:** 2014-04-23

**Authors:** Vassilis Mavromatidis, Zoltan Varga, Frigyes Waczek, Zoltán Őrfi, László Őrfi, György Kéri, George Mosialos

**Affiliations:** 1 School of Biology, Aristotle University of Thessaloniki, Thessaloniki, Greece; 2 Vichem Chemie Ltd., Budapest, Hungary; 3 Department of Molecular Biology, Max-Planck-Institute of Biochemistry, Martinsried, Germany; 4 Department of Pharmaceutical Chemistry, Semmelweis University, Budapest, Hungary; 5 MTA-SE Pathobiochemistry Research Group, Department of Medical Chemistry, Semmelweis University, Budapest, Hungary; University of Nebraska – Lincoln, United States of America

## Abstract

Epstein-Barr virus (EBV) is a human herpesvirus, which is causally associated with the development of several B lymphocytic malignancies that include Burkitt's lymphomas, Hodgkin's disease, AIDS and posttransplant associated lymphomas. The transforming activity of EBV is orchestrated by several latent viral proteins that mimic and modulate cellular growth promoting and antiapoptotic signaling pathways, which involve among others the activity of protein kinases. In an effort to identify small molecule inhibitors of the growth of EBV-transformed B lymphocytes a library of 254 kinase inhibitors was screened. This effort identified two tyrosine kinase inhibitors and two MEK inhibitors that compromised preferentially the viability of EBV-infected human B lymphocytes. Our findings highlight the possible dependence of EBV-infected B lymphocytes on specific kinase-regulated pathways underlining the potential for the development of small molecule-based therapeutics that could target selectively EBV-associated human B lymphocyte malignancies.

## Introduction

Epstein-Barr virus (EBV) is a human γ-herpesvirus that infects B lymphocytes as well as epithelial cells. Latent EBV infection is associated with several malignancies of B lymphocytes that include Burkitt's lymphomas, Hodgkin's lymphomas, AIDS-associated lymphomas, and post-transplantation lymphoproliferative disorders (PTLDs). *In vitro*, EBV can transform resting B cells into long-term proliferating lymphoblastoid cell lines (LCLs). LCLs display type III latency, in which a limited set of viral genes is expressed. This set includes six EBV nuclear antigens (EBNA 1, 2, 3A, 3B, 3C and-LP), three membrane proteins (LMP1, LMP2A and LMP2B), two short non-polyadenylated RNAs (EBER1 and EBER2) and transcripts from the BamHI A region of EBV (BARTs) (reviewed in [1]). The process of B lymphocyte transformation by EBV relies on the usurpation of cellular growth promoting and antiapoptotic signaling pathways by viral proteins. For example, LMP2A mimics and modulates the signaling pathway of B-cell antigen receptor (BCR) whereas the LMP1 oncoprotein mimics a constitutively active CD40 receptor [2], [3]. These signaling pathways involve a number of protein kinases, which are functionally modulated by the activity of latent EBV antigens.

BCR is composed of a tetrameric complex of immunoglobulin (Ig) polypeptides (two heavy and two light chains) in association with Igα (CD79A) and Igβ (CD79B) molecules. Igα and Igβ contain a cytoplasmic immunoreceptor tyrosine-based activation motif (ITAM) [4]. ITAMs transmit signals that lead to the activation of several protein tyrosine kinases (PTKs), including those of Src family (such as Fyn, Lyn, Lck and cSrc), spleen tyrosine kinase (Syk) and Bruton's tyrosine kinase (Btk) [5], [6]. This BCR signaling through PTKs has been shown to be essential for the survival of a number of murine and human B-cell lymphoma cell lines [7]. Importantly, LMP2A protein harbors an ITAM in its amino-terminal cytoplasmic domain. In EBV-transformed LCLs, the amino-terminal domain of LMP2A is constitutively phosphorylated on tyrosine residues and is associated with PTKs, such as Lyn and Syk [8]. LMP2A can block BCR signal transduction and prevent the activation of lytic replication of EBV in LCLs thus maintaining virus latency [9]–[11]. Moreover, LMP2A can act as a BCR mimic, since human B cells, which do not express functional BCR, are rescued from apoptosis when are infected with wild type EBV, but not with EBV lacking LMP2A [12]. In accordance with this, studies in transgenic mice that express LMP2A in B cells have shown that LMP2A expression can promote survival in BCR-negative cells [13], [14].

Apart from PTKs, the Raf/MEK/ERK pathway has been implicated in transformed B cell survival although the role of this pathway in the survival of EBV-transformed human B cells is unclear. ERKs can be activated by either LMP1 or LMP2A among the latent EBV antigens [15], [16]. Interestingly, LMP1-mediated Erk activation is essential for Rat1 transformation by LMP1 [15]. Furthermore, sustained Erk activation by LMP2A can provide an essential survival function in developing B cells [17]. These findings support a prosurvival role of the ERK pathway in B lymphocyte transformation by EBV.

Given their crucial role in the development of B-cell malignancies, protein kinases are potential targets for anticancer therapies [18], [19]. Protein kinase inhibitors (PKIs) are useful tools for this strategy and have already been used as drugs against several types of cancer (reviewed in [20]). In this study, a library of 254 PKIs was screened in order to identify inhibitors that show specific activity against EBV-infected B cells. Importantly, two tyrosine kinase inhibitors, as well as two MEK inhibitors, out of the 254 tested, were found to impair more potently the viability of EBV-infected cells compared to non-infected B cells. These results indicate that the impact of inhibition of specific protein kinases in cell viability differs among B lymphocytes in a manner that may depend on latent EBV infection. Consequently, the use of specific kinase inhibitors may prove to be beneficial for the treatment of EBV-related lymphomas.

## Materials and Methods

### Ethics Statement

The use of human samples was approved by the responsible Institutional Review Board (George Papanicolaou Hospital Board of Directors, reference number: ΑΔΑ Β43346906Β-Σ6Ε). The participants provided their written informed consent to participate in this study.

### Inhibitors

The 254 PKIs that were tested in this study constitute part of the Chemical Validation Library (CVL), which is the core library of the Nested Chemical Library of Vichem and consists of launched kinase inhibitor drugs and compounds in clinical trials and in preclinical development (http://www.vichem.hu/nested_chemical_library.html) [21]. Four of these 254 PKIs were further studied; PP2 is an inhibitor of Src family tyrosine kinases with higher potency for Lck and Fyn. Compound 5 also inhibits Src family kinases with Lck being the most sensitive. CI-1040 and PD 198306 are MEK inhibitors. PP2, CI-1040 and PD 198306 have already been used in pharmacological studies [22]–[24]. Inhibitors were synthesized according to literature procedures: PP2 [25], compound 5 [26], CI-1040 and PD 198306 [27]. A-770041 (Axon Medchem) is a selective inhibitor of Lck, while U0126 (Gibco) is a selective inhibitor of MEK1 and MEK2. All inhibitors were dissolved in DMSO.

### Cell lines and tissue culture reagents

LCL-WT [28] and LCL-FLAG-LMP1 [29] are EBV transformed lymphoblastoid cell lines. BL41-B95-8 [30] is an EBV-positive (EBV+) Burkitt's lymphoma cell line, while BL41 [31] and DG75 [32] are EBV-negative (EBV-) Burkitt's lymphoma cell lines. Human peripheral blood mononuclear cells (PBMCs) from healthy donors were isolated using Histopaque-1077 (Sigma-Aldrich), according to the instructions of the manufacturer. B cell lines and PBMCs were cultured in RPMI1460 (Invitrogen) supplemented with 10% fetal bovine serum, L-glutamine (2 mM), penicillin (100 units/mL) and streptomycin (0.1 mg/mL).

### Viability assay

B cells or PBMCs were seeded in 12 well plates (day 0). Different concentrations of each inhibitor were added in the cultures at days 0 and 2. Cell viability was measured at day 4 either by trypan blue exclusion or using MTT assay and expressed as percent survival, which was calculated by comparing the viability of treated vs DMSO treated cells and assigning the latter the value of 100%. IC_50_s were calculated in GraphPad Prism software using a four parameters fitting. Top and Bottom constrains were set to 100 and 0. Values with R^2^>0.8 regression are displayed in Table 1.

**Table 1:** The IC_50_ values of the kinase inhibitors towards B lymphoma cell lines and PBMCs are shown.

	IC_50_ values (µM)			
	PP2	Compound 5	CI-1040	PD 198306
**LCL-WT**	0.75	<0.05	1.00	1.89
**LCL-FLAG-LMP1**	1.12	<0.05	0.41	0.96
**DG75**	5.81	>2	4.10	3.48
**PBMCs**	11.04	>8	7.92	13.72
**BL41-B95-8**	2.45	<0.2	1.77	0.61
**BL41**	7.40	>4	4.00	2.87

### Apoptosis assay

B cells were seeded in 12 well plates at a concentration of 10^5^ cells/ml and each inhibitor was added in two doses, at days 0 and 2 after seeding. One day after the last dose, cells were harvested, stained with Annexin V-FITC (eBioscience) and propidium iodide (PI, eBioscience), according to the instructions of the manufacturer, and the percentage of the early apoptotic cells was measured using flow cytometry.

### Western blot analysis

10^5^ B cells/ml were seeded in 6 well plates and incubated with inhibitors for up to 48 hours. Cells (at least 5×10^5^) were harvested at specific time points and whole extracts were isolated by boiling the cells in sodium dodecyl sulfate polyacrylamide gel electrophoresis (SDS-PAGE) loading buffer for 10 minutes. Cell isolates were analyzed by SDS-PAGE, followed by electrophoretic transfer and immunoblotting. Antibodies against phospho-ERK (E-4; Santa Cruz Biotechnology), ERK 2 (C-14; Santa Cruz Biotechnology), β-actin (C4; Santa Cruz Biotechnology) and tyrosine kinase substrates (4G10 anti-phosphotyrosine) were used for Western blot analysis.

### RNA interference and electroporation assay

The expression of *Lck* and *MEK1* genes was downregulated using short hairpin RNA (shRNA) expression vectors. These vectors were constructed by cloning appropriate oligonucleotides into the pHEBo-SUPER plasmid [33] between the unique BglII and HindIII sites of its polylinker. The oligonucleotide sequences that were used were the following: LCK(A)(for); 5′- GATCCCCACGGAATTATATTCATCGTGACTTCAAGAGAGTCACGATGAATATAATTCCGCTTTTTA- 3′ and LCK(A)(rev); 5′- AGCTTAAAAAGCGGAATTATATTCATCGTGACTCTCTTGAAGTCACGATGAATATAATTCCGTGGG-3′ for the construction of the pHEBo-Lck(A) shRNA-expression vector against LCK, LCK(B)(for); 5′- GATCCCCCCCTGGACATGGCAGCCCAAATTTCAAGAGAATTTGGGCTGCCATGTCCAGGATTTTTA- 3′ and LCK(B)(rev); 5′- AGCTTAAAAATCCTGGACATGGCAGCCCAAATTCTCTTGAAATTTGGGCTGCCATGTCCAGGGGGG-3′ for the construction of the pHEBo-Lck(B) shRNA-expression vector against LCK, MEK1A(for); 5′-GATCCCCCGGTCCTACATGTCGCCAGAAATTCAAGAGATTTCTGGCGACATGTAGGACCTTTTTTA-3′ and MEK1A(rev); 5′- AGCTTAAAAAAGGTCCTACATGTCGCCAGAAATCTCTTGAATTTCTGGCGACATGTAGGACCGGGG-3′ for the construction of the pHEBo-MEK1(A) shRNA-expression vector against MEK1, and MEK1B(for); 5′- GATCCCCCGGTCATGGCCAGAAAGCTAATTTCAAGAGAATTAGCTTTCTGGCCATGACCATTTTTA-3′ and MEK1B(rev); 5′- AGCTTAAAAATGGTCATGGCCAGAAAGCTAATTCTCTTGAAATTAGCTTTCTGGCCATGACCGGGG-3′ for the construction of the pHEBo-MEK1(B) shRNA-expression vector against MEK1. 3×10^6^ cells of LCL-WT or LCL-FLAG-LMP1 were suspended in 250 µL Opti-MEM I Reduced Serum Medium (Gibco). Cells were electroporated with 5 µg pMAX-GFP and 10 µg pHEBo-SUPER at 140 V and 1000 µF in a 0.2 cm cuvette (Bio-Rad) and then transferred in 1.5 ml culture medium. shRNAs against GAPDH and luciferase gene were used as negative and positive control respectively. Two days after electroporation the percentage of transfected cells was determined *via* the detection of green fluorescent protein (GFP) using flow cytometry, and 2×10^5^ of the cells were placed in 1.5 ml medium supplemented with hygromycin at the concentration of 200 µg/ml. Cell viability was measured using the MTT assay.

### Statistical analysis

Data are presented as means ± standard error of the mean (SEM). The statistical evaluation was performed using Student's *t*-test. All statistical analyses were performed with Statistica 5.0. p values of ≤.05 were considered significant.

## Results

### Tyrosine kinase inhibitors and ERK inhibitors exert a selective negative effect on the viability of EBV-infected B cells

**Figure 1:**
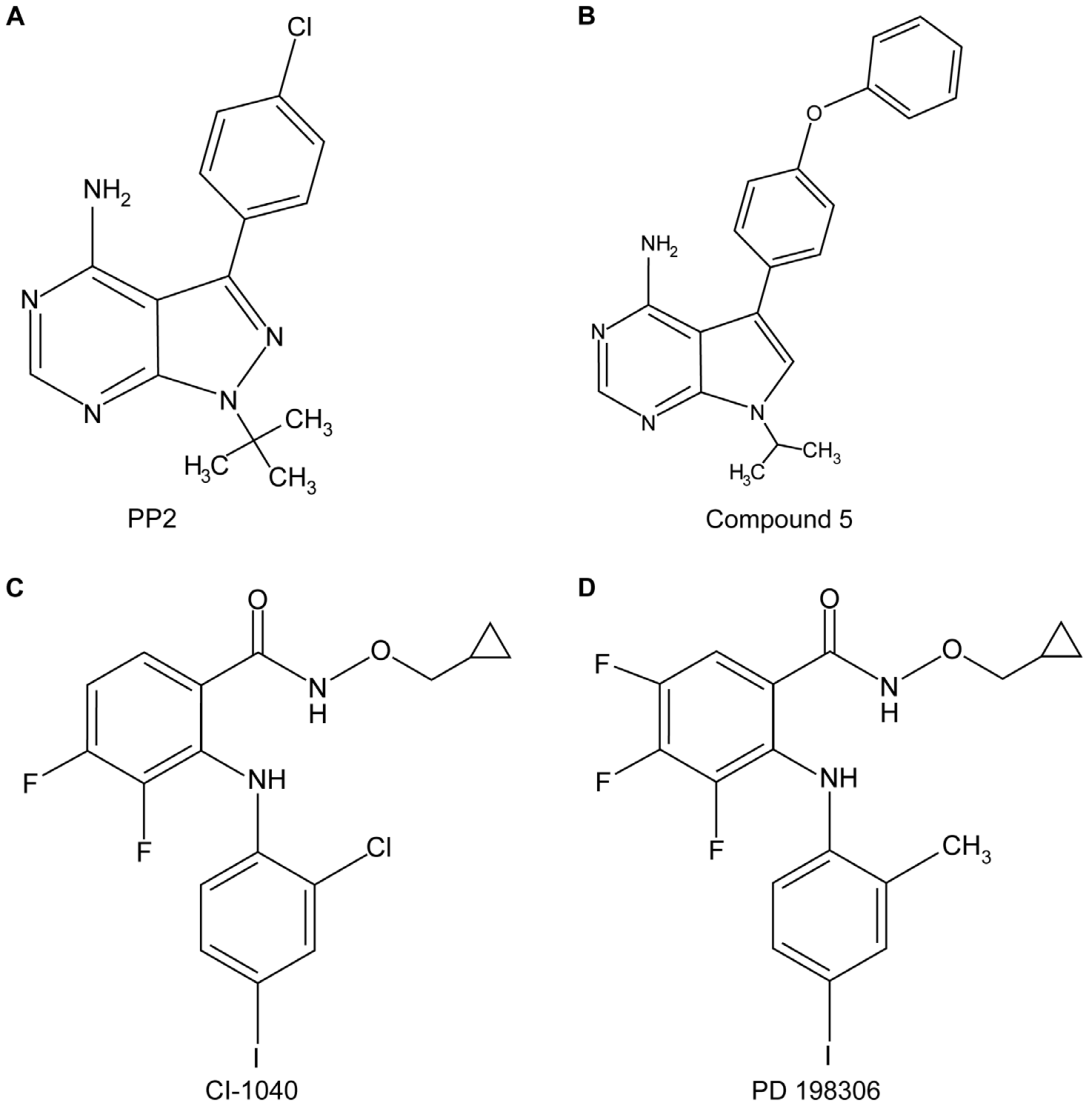
The structure of kinase inhibitors used in this study. PP2 (A) and compound 5 (B) are Src-family tyrosine kinases inhibitors, while CI-1040 (C) and PD 198306 (D) are MEK inhibitors.

In order to find kinase inhibitors that affect selectively the viability of cancer B cell lines that are infected with Epstein-Barr virus (EBV), a library of 254 low molecular weight kinase inhibitors (part of Chemical Validation Library of Vichem), was screened using cells that are either infected or non-infected by EBV. More specifically, LCL-WT and DG75 were treated with 1 µM of each inhibitor for 4 days and then the cell viability was evaluated by trypan blue exclusion (Figure S1). Compounds that inhibited the viability of LCL-WT by at least 50% but did not reduce the viability of DG75 cells by more than 50% were tested further for their effect against an additional EBV-transformed B cell line (LCL-FLAG-LMP1) and normal peripheral blood mononuclear cells (PBMCs). Four compounds were found to compromise the viability of EBV-positive cells preferentially (Figure S1). Two of these compounds are Src family tyrosine kinase inhibitors (PP2 and compound 5), while the other two compounds inhibit primarily the ERK pathway through the inhibition of MEK kinase (CI-1040 and PD 198306) (Figure 1).

**Figure 2:**
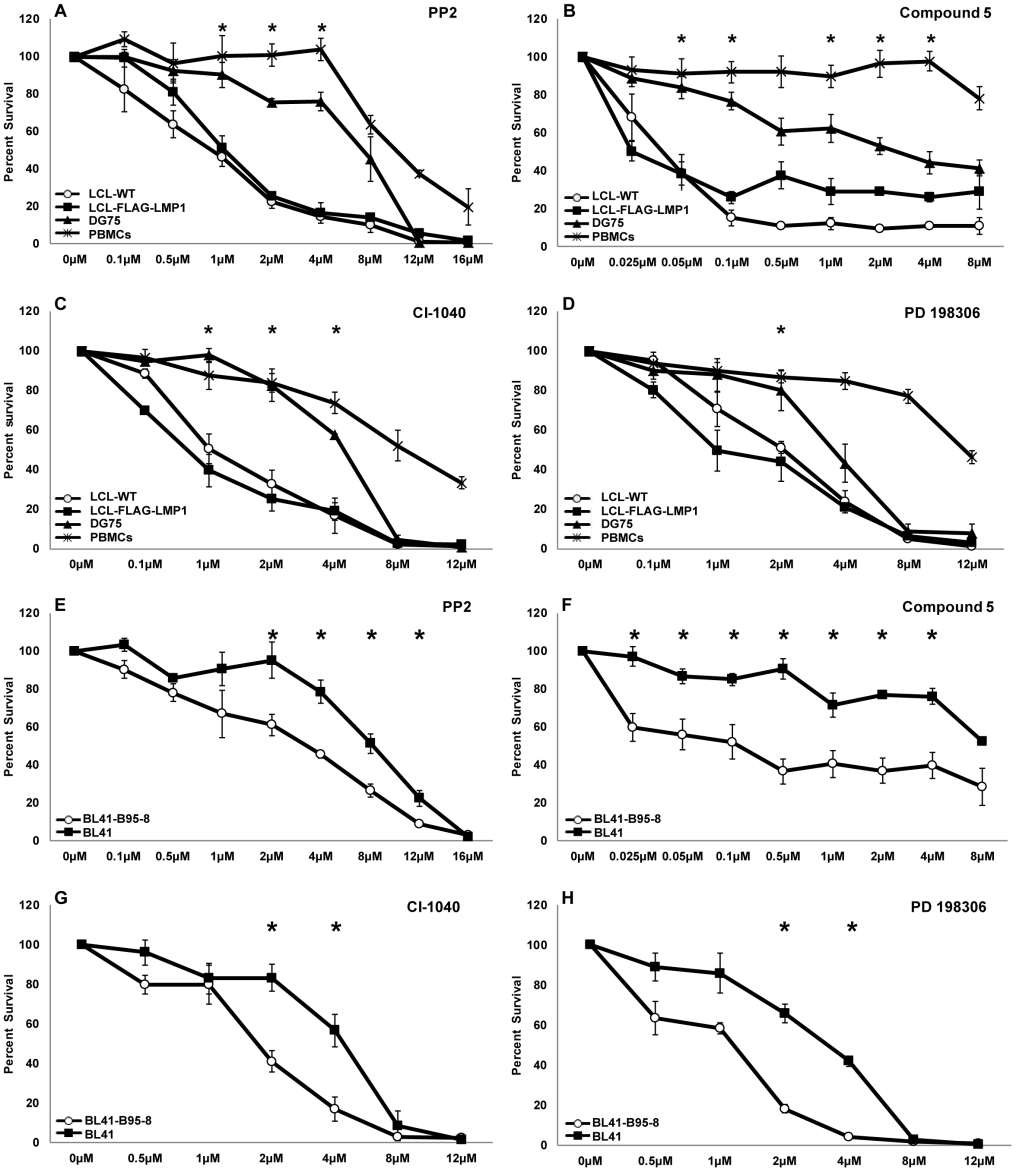
The effect of kinase inhibitors on B cell and PBMC viability. The viability curves of B cells and PBMCs after four days of incubation with different concentrations of each inhibitor are depicted. Treatment of cells with DMSO served as control. (A–D): Percent survival of LCL-WT (open circles), LCL-flag-LMP1 (solid rectangles), DG75 (solid triangles) and PBMCs (asterisks) treated with inhibitor PP2 (A), compound 5 (B), CI-1040 (C) or PD 198306 (D). (E–H): BL41-B95-8 (open circles) and BL41 cell line (solid rectangles) percent survival after treatment with inhibitor PP2 (E), compound 5 (F), CI-1040 (G) and PD 198306(H). The results are the means ± SEM from three independent experiments. Statistical analysis was performed using Student's *t* test. Statistically significant differences (p<0.05) in viability between each LCL and DG75 as well as PBMCs (panels A to D), or between BL41-B95-8 and BL41 cell lines (panels E to H) are shown by *.

In order to analyze further the action of these four inhibitors, their dose-dependent effect on the viability of LCL-WT, LCL-FLAG-LMP1, DG75 and PBMCs was determined. Of note, a greater reduction in the viability of EBV+ cell lines compared to EBV- cell lines and PBMCs was observed in all cases (Figure 2A–D). There was at least one concentration of each inhibitor that caused a statistically significant difference in cell viability between EBV+ and EBV- cells. The IC_50_ values of PP2, Cl-1040 and PD 198306 are presented in Table 1. In the case of compound 5 approximate IC_50_ values are shown due to the slope of the curves (r^2^<0.8).

In order to evaluate further the selectivity of the inhibitors towards the viability of EBV+ cells, the compounds were tested against additional cell lines. BL41 is an EBV-negative Burkitt's lymphoma cell line, while BL41-B95-8 has been derived by the infection of BL41 with the B95-8 strain of EBV. Treatment of these cell lines with the kinase inhibitors reduced the viability of BL41-B95-8 cells to a greater extent compared to their parental EBV- (BL41) cells (Figure 2E–H). The differential effect of the inhibitors towards the two cells lines is also evident by the corresponding IC_50_ values (Table 1). A similar pattern of results was obtained by evaluating cell viability using the MTT assay (data not shown).

### The reduction in EBV-infected B cell viability by kinase inhibitors correlates with apoptosis initiation

**Figure 3:**
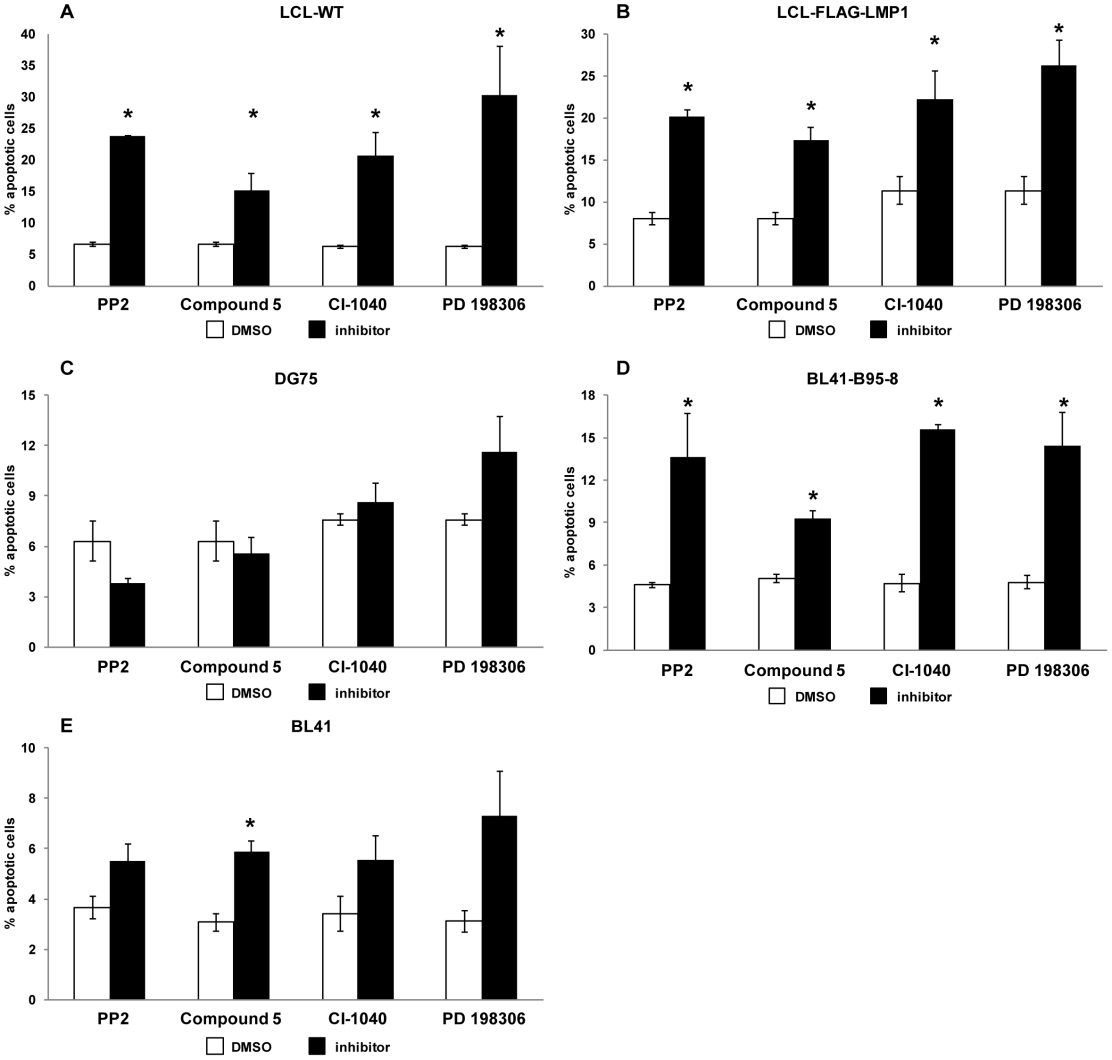
Apoptosis evaluation in B cell lines treated with kinase inhibitors. Cells were treated with PP2 (8 µM), compound 5 (0.05 µM for LCL-WT, LCL-flag-LMP1 and DG75 or 1 µM for BL41-B95-8 and BL41), CI-1040 (4 µM) or PD 198306 (4 µM) for 72 hours and the apoptotic index was assessed by Annexin V - PI staining. The percentage of early apoptotic (AnnexinV+PI-) LCL-WT (A), LCL-flag-LMP1 (B), DG75 (C), BL41-B95-8 (D) or BL41 (E) cells treated with the indicated kinase inhibitor (black column) or left untreated (white column) is shown. Results are the means ± SEM from at least three independent experiments. Statistical analysis was performed using Student's *t* test. Statistically significant differences (p<0.05) between treated and untreated cells are shown by *.

The basis for B cell viability reduction was investigated in order to understand the mechanism of action of the kinase inhibitors. For this purpose, each cell line that was used in the viability assay (Figure 2) was incubated with each one of the kinase inhibitors for 72 h and the cells were subsequently analyzed for evidence of apoptosis using Annexin V and PI staining. Notably, all inhibitors caused a statistically significant increase in the percentage of early apoptotic cells, as assessed by Annexin V - PI staining, in all the EBV-infected cell lines (LCL-WT, LCL-FLAG-LMP1 and BL41-B95-8) without affecting significantly the apoptotic profile of the EBV- cell lines (DG75 and BL41) (Figure 3). More specifically, treatment with the tyrosine kinase inhibitor PP2 or compound 5 induced an increase in the percentage of early apoptotic cells in LCLs from up to 8% to at least 20% and 15% respectively (Figures 3A and 3B). Similarly, the CI-1040 and PD 198306 inhibitors caused an increase in the percentage of early apoptotic cells in LCLs from up to 11% to at least 20% and 25% respectively (Figures 3A and 3B). Likewise, there was an increase in the percent of early apoptotic BL41-B95-8 cells as a result of treatment with PP2 (from 4.5% to 13%), compound 5 (from 5% to 9%), CI-1040 (from 5% to 15%) or the PD 198306 (from 5% to 15%) inhibitor (Figure 3D). On the contrary, the extent of apoptosis in the EBV- cells DG75 (Figure 3C) and BL41 (Figure 3E) was not affected significantly after their incubation with each of the inhibitors under the conditions specified above, with the exception of compound 5 which caused a statistically significant increase in the apoptosis of BL41 cells. These findings indicate that the reduction in cell viability by the kinase inhibitors that were used was mediated at least in part, by induction of apoptosis. Clearly, other processes of cell death could contribute to the reduction in cell line viability by the PKIs used.

### Targets of protein kinase inhibitors that negatively affect the viability of EBV-infected B cells

**Figure 4:**
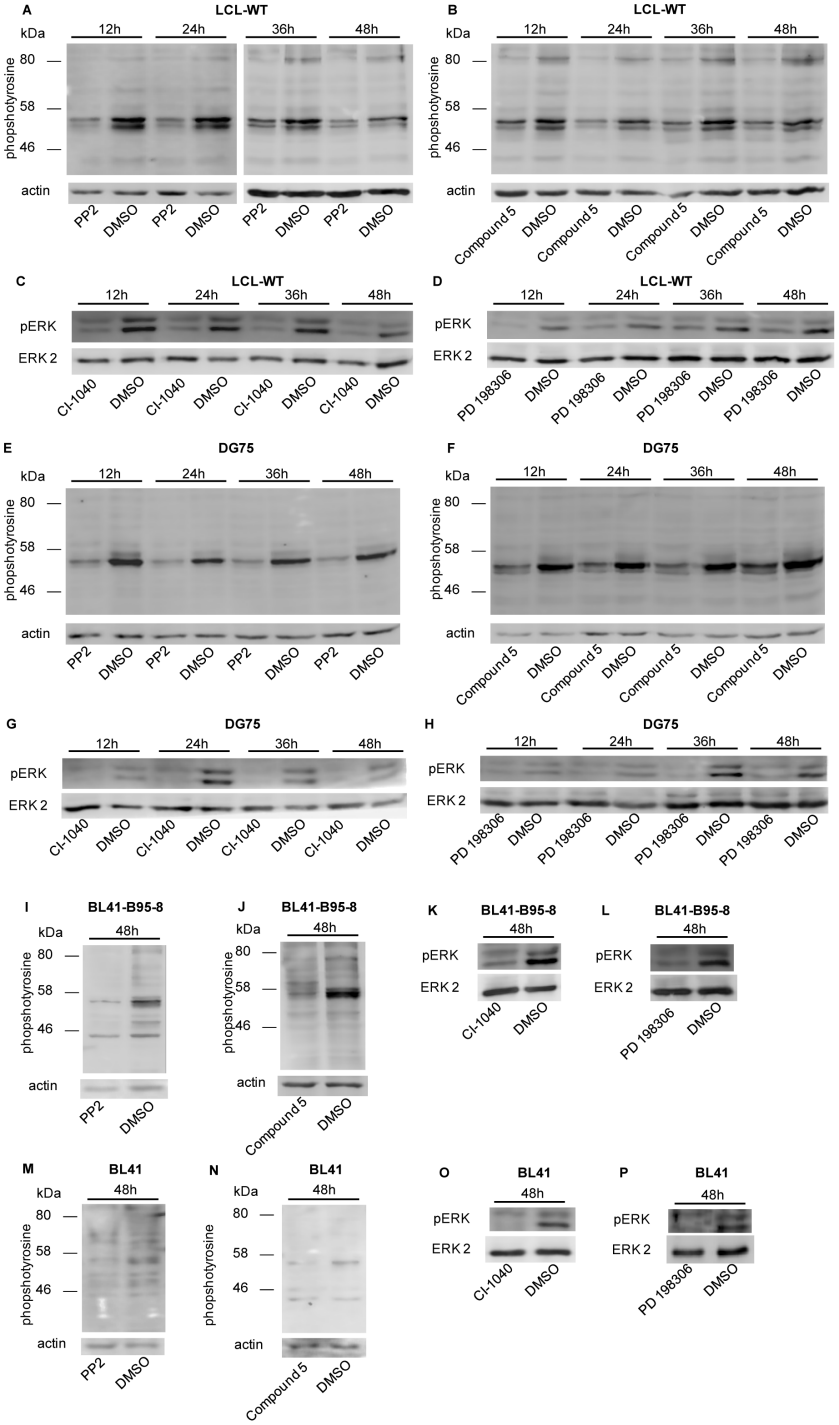
Effect of kinase inhibitors on tyrosine phosphorylation or ERK phosphorylation in B cell lines. LCL-WT and DG75 cell lines were treated with PP2 (8 µM), compound 5 (0.05 µM), CI-1040 (4 µM) or PD 198306 (4 µM), while BL41-B95-8 and BL41 were treated with PP2 (2 µM), compound 5 (0.5 µM), CI-1040 (2 µM) or PD 198306 (2 µM) for up to 48 hours. Treatment of cells with DMSO served as control. Whole cell lysates were prepared at specific time points and protein tyrosine phosphorylation or ERK-1 and -2 phosphorylation was evaluated using immunoblotting. Whole cell extracts obtained from LCL-WT (A–D), DG75 (E–H), BL41-B95-8 (I–L) or BL41 (M–P) after treatment with the compounds PP2 and compound 5, or CI-1040 and PD 198306 were immunoblotted for phosphotyrosine or phospho-ERK as indicated. Results are representative of two independent experiments.

PP2 and compound 5 are small molecule inhibitors of Src family tyrosine kinases, whereas CI-1040 and PD 198306 inhibit primarily the activation of MEK/ERK pathway. To analyze the molecular mechanisms that may be responsible for the selectivity of kinase inhibitors towards EBV-infected B cells, the levels of proteins with phosphorylated tyrosine residues and phosphorylated ERKs were examined in the EBV+ LCL-WT and the EBV- DG75 cells that were treated with each inhibitor for up to 48 hours. Cell extracts were isolated every 12 hours and the levels of phospho-tyrosine as well as phospho-ERK were determined by immunoblotting. Both PP2 and compound 5 inhibited tyrosine phosphorylation of several proteins in the EBV+ LCL-WT (Figure 4A and 4B) as well as in the EBV- DG75 cells (Figure 4E and 4F) during the treatment period. In addition, we assessed the levels of phospho-tyrosine in cell extracts, derived from Burkitt's lymphoma cell lines, after treatment with PP2 or compound 5 for 48 hours. Indeed, both the BL41-B95-8 (EBV+) (Figure 4I and 4J) and the BL41 (EBV-) cell extracts (Figure 4M and 4N) exhibited reduced tyrosine phosphorylation after treatment compared with the untreated cells. It should be noted that in addition to similar-size phosphotyrosine substrates, EBV+ cell extracts contained also tyrosine-phosphorylated proteins not seen in the EBV- cell extracts. In the case of the CI-1040 and PD 198306 compounds, extensive and comparable reduction in the levels of phosphorylated ERK1-2 was observed in both LCL-WT (Figures 4C and 4D) and DG75 cells (Figures 4G and 4H) at each time point tested. Similar results were obtained in the BL41-B95-8 (Figures 4K and 4L) and BL41 cell lines (Figures 4O and 4P). These data suggest that inhibition of tyrosine phosphorylation of common and/or distinct proteins by PP2 and compound 5 or ERK1-2 by CI-1040 and PD 198306, may contribute to the higher viability reduction of EBV-infected B cells compared to the non-infected ones.

**Figure 5:**
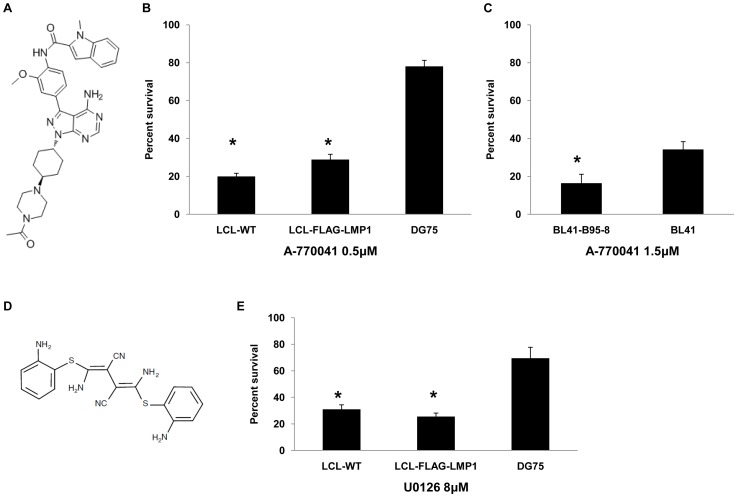
Effect of A-770041 and U0126 on B lymphoma cell viability. (A) Structure of Lck inhibitor A-770041. (B) Percent of viable cells of LCL-WT, LCL-FLAG-LMP1 and DG75 after treatment with 0.5 µM of A-770041 for 4 days. The results are the means ± SEM from three independent experiments. Statistically significant (p<0.05) differences between the viability of each LCL cell line and DG75 are shown by an asterisk. (C) Percent of viable cells of BL41-B95-8 and BL41 after treatment with 1.5 µM of A-770041 for 4 days. The results are the means ± SEM from three independent experiments. Statistically significant (p<0.05) differences in viability between these two cell lines is denoted by an asterisk. (D) Structure of MEK inhibitor U0126. (E) Percent of viable cells of LCL-WT, LCL-FLAG-LMP1 and DG75 after treatment with 8 µM of U0126 for 4 days. The results are the means ± SEM from three independent experiments. Statistically significant (p<0.05) differences between the viability of each LCL cell line and DG75 are denoted by an asterisk.

Since a common target of PP2 and compound 5 is Lck, we investigated whether another Lck inhibitor could mimic their effect on B lymphoma cell lines. A-770041, a known selective inhibitor of Lck (Figure 5A), was used in order to test the influence of Lck inhibition on the viability of EBV+ and EBV- lymphocytes. Treatment of LCL-WT, LCL-FLAG-LMP1 and DG75 cell lines with 0.5 µM of A-770041 inhibitor for 4 days caused a higher reduction in the viability of the two LCLs in relation to DG75 (Figure 5B). Similarly, treatment of BL41-B95-8 or BL41 with 1.5 µM of A-770041 caused a more prominent reduction in the viability of BL41-B95-8 compared to BL41 cells (Figure 5C). These experiments suggest that inhibition of Lck may be the basis, at least in part, for the selective negative effect of PP2 and compound 5 on the viability of EBV-positive B cells.

In order to investigate further the role of MEK in the viability of EBV+ B cells the effect of U0126, a selective MEK inhibitor (Figure 5D) was evaluated. LCL-WT, LCL-FLAG-LMP1 and DG75 cell lines were incubated with 8 µM of U0126 for 4 days. This treatment resulted in a negative effect on the viability of the cells, which was more potent for LCLs than DG75 cells (Figure 5E). This result underscores the greater dependence of EBV-infected B cells on the activity of MEK in comparison to non-infected B cells.

**Figure 6:**
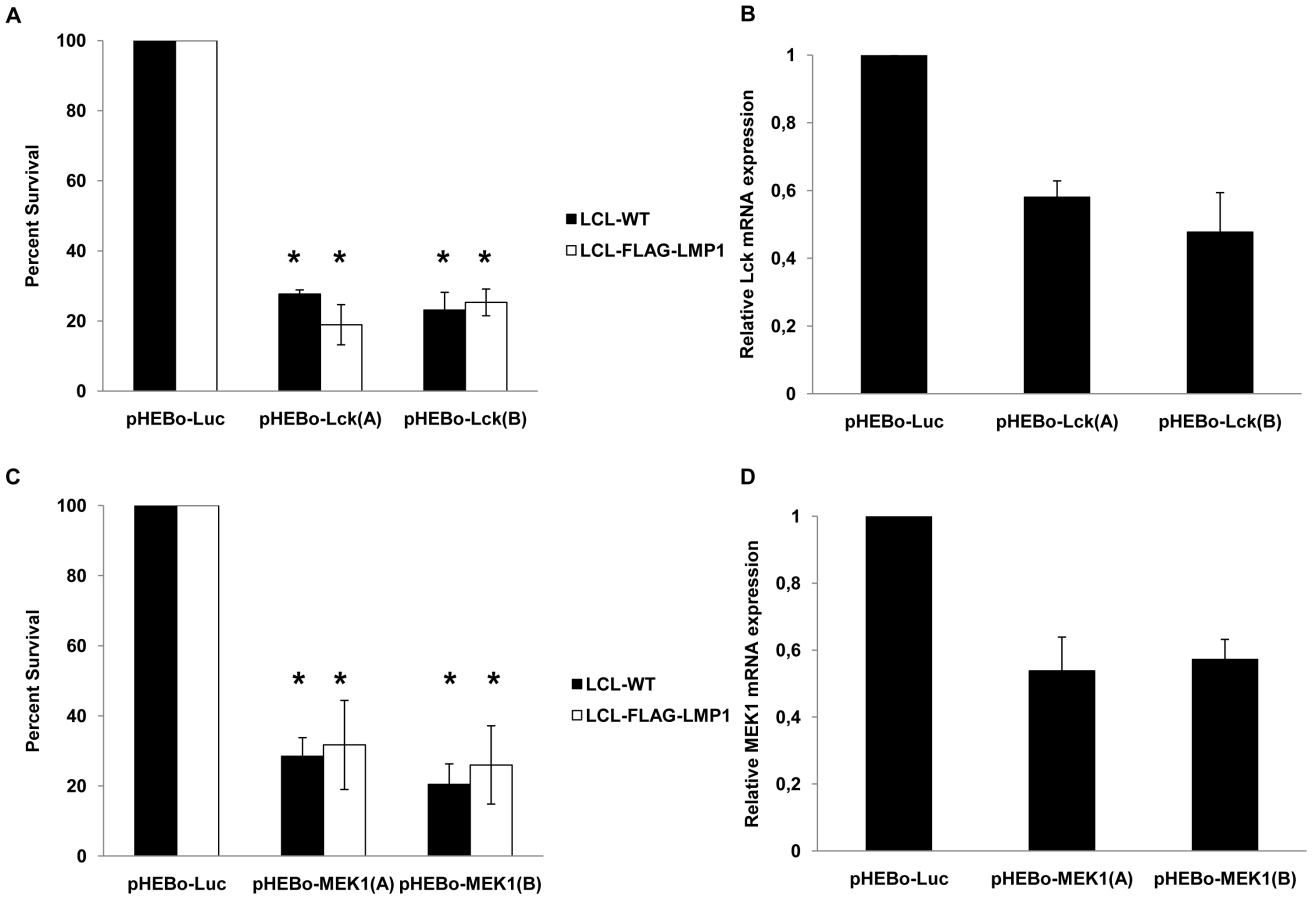
Effect of LCK and MEK1 downregulation on LCL viability. The viability of EBV-transformed LCL was assessed following the expression of shRNAs against Lck (A) or MEK1 (C) in LCL-WT (black bars) and LCL-FLAG-LMP1 cells (white bars). Cells transfected with shRNA-expression vectors against luciferase gene were used as reference control. The results are the means ± SEM from three independent experiments. Differences in viability between transfected cells with pHEBo-Luc and pHEBo-LCK or pHEBo-MEK1 vectors are statistically significant (p<0.05). (B and D) Downregulation of *Lck* (B) or *MEK1 (D)* mRNA expression by pHEBo vectors. 293FT cells were transfected with pHEBo-Lck (2 µg) or pHEBo-MEK1 (2 µg) or pHEBo-Luc (2 µg). RNA extracts were isolated two days after transfection and the levels of the expression of *Lck* or *MEK1* gene were estimated by RT-PCR. The results from a representative experiment out of two independent experiments are shown.

The role of Lck and MEK1 in the viability of LCLs was studied further by using shRNAs that silence these two genes. Vectors expressing two shRNAs that target *Lck* and two shRNAs that target *MEK1* were electroporated in either LCL-WT or LCL-FLAG-LMP1 cells. After selection with hygromycin, LCL-WT cells that were transfected with these plasmids exhibited at least 71% reduction of their viability compared with cells that were transfected with shRNA against an irrelevant gene (luciferase). Similar results were observed in LCL-FLAG-LMP1 cells, as the reduction of cells' viability was at least 68% (Figure 6).

Taken together these results indicate that the inhibition of Lck and MEK1 may account, at least partly, for the greater sensitivity of EBV+ versus EBV- B cells to the inhibitors that were identified by this study.

## Discussion

Protein kinases play a vital role in the survival, development and proliferation of B cells. Hence, protein kinase inhibitors (PKI) have been used for treatment in patients with neoplastic and chronic inflammatory diseases. Since there are several agents, such as the Hsp90 inhibitor 17-DMAG [34] and simvastatin [35], that have been found to affect selectively the viability of EBV-infected cells, we examined whether there are PKIs with similar function. Indeed, two tyrosine kinase inhibitors, PP2 and compound 5, and two MEK inhibitors, CI-1040 and PD 198306, showed a selective negative effect towards EBV+ cells.

Treatment with PP2 or compound 5 caused a significant reduction in the phosphorylation level of specific substrates of all tested cell lines, as it was found by immunoblotting (Figure 4). This analysis revealed that a major target of PP2 and compound 5 was a protein with molecular weight of approximately 55 kDa. Considering Src family kinases that participate in B cell viability, one possible candidate for the 55 kDa molecule, could be the kinase Lck. Lck is predominantly expressed in T cells and is undetectable in peripheral blood B cells from healthy donors. However, it is expressed in normal B cells following transformation by EBV [36], in chronic lymphocytic leukemia (B-CLL) [37], in LCLs and in BL cell lines [38]. Interestingly, Lck can phosphorylate the cytoplasmic tail of both Igα and Igβ *in vitro* [39]. Since PP2 and compound 5 were the only two inhibitors of Chemical Validation Library (CVL) out of 254 tested that inhibit Lck, we tested the possibility that Lck is a critical mediator of the selective effect of these two inhibitors towards EBV+ B cells. A-770041 is a selective inhibitor of Lck that has different molecular structure from PP2 and compound 5 [40]. Similarly to PP2 and compound 5, A-770041 caused a higher reduction in the viability of EBV+ cells in comparison to EBV- cells (Figure 5B and 5C). Moreover, RNA interference experiments revealed that Lck kinase is essential for the viability of LCLs, since downregulation of *Lck* expression had a potent negative effect on their viability (Figure 6A). In summary, these data indicate that the selective negative effect of PP2 and compound 5 on EBV+ positive B cells may be mediated, at least in part, by Lck inhibition.

CI-1040 and PD 198306 are the only two inhibitors of CVL compounds that inhibit ERK1/2 phosphorylation. Deregulation of the MEK/ERK pathway has been associated with sensitivity and resistance to leukemia therapy highlighting the importance of this pathway for controlling hemopoietic malignancies [41]. U0126 is a selective MEK inhibitor, structurally dissimilar compared to CI-1040 and PD 198306 [42]. Treatment with U0126 led to increased cell death of LCLs compared to DG75 (Figure 5E). However, the pair of BL41 and BL41-B95-8 cell lines didn't exhibit similar results. BL41-B95-8 cells weren't more sensitive than BL41 to U0126 (data not shown). Recently it was found that U0126 and other MEK inhibitors have off-target effects, independent of their ability to inhibit MEK1/2 [43]. This finding could explain the similar influence of U0126 on the viability of BL41 and BL41-B95-8 cell lines.

Although our experiments indicate that Lck or MEK kinase inhibition contributes to the selective negative effect of the four inhibitors on EBV+ B cells, it should be pointed out that additional effects of the four inhibitors on unknown kinases may shape as well their overall effect towards EBV+ B cells. In addition, it should be stressed that although our data clearly indicate a differential sensitivity between EBV+ and EBV- B cells to the four kinase inhibitors identified by our screen, this notion cannot be fully generalized since polymorphisms, mutations and other types of chromatin modifications may alter the response of individual B lymphoma cells to the inhibitors used. Nevertheless, our results have identified four kinase inhibitors that could provide the structural basis for the development of small molecule therapeutics with selective activity against EBV-associated B cell malignancies.

## Supporting Information

Figure S1 **Initial screening of the Chemical Validation Library.** LCL- WT and DG75 cells were treated with each one of 254 kinase inhibitors (1 µM) of the Chemical Validation Library for 4 days. The results are the means from three independent experiments. Compounds that inhibited the viability of LCL-WT by at least 50% but did not reduce the viability of DG75 cells by more than 50% were tested further for their effect against an additional EBV-transformed B cell line (LCL-FLAG-LMP1) and PBMCs. The four inhibitors (PP2, compound 5, CI-1040 and PD 198306) that were found to compromise the viability of EBV-positive cells preferentially and analyzed in the present study are indicated by arrows. The rest of the inhibitors of the upper left quadrant were not analyzed further because they did not exhibit similar results, when tested against LCL-FLAG-LMP1 and PBMCs.
(TIF)
