# Supplementary figures and images for: Identification of Protein Kinase Inhibitors with a Selective Negative Effect on the Viability of Epstein-Barr Virus Infected B Cell Lines
Source: PLoS One. 2014 Apr 23;9(4):e95688. doi: 10.1371/journal.pone.0095688 (PMC3997413; doi:10.1371/journal.pone.0095688)

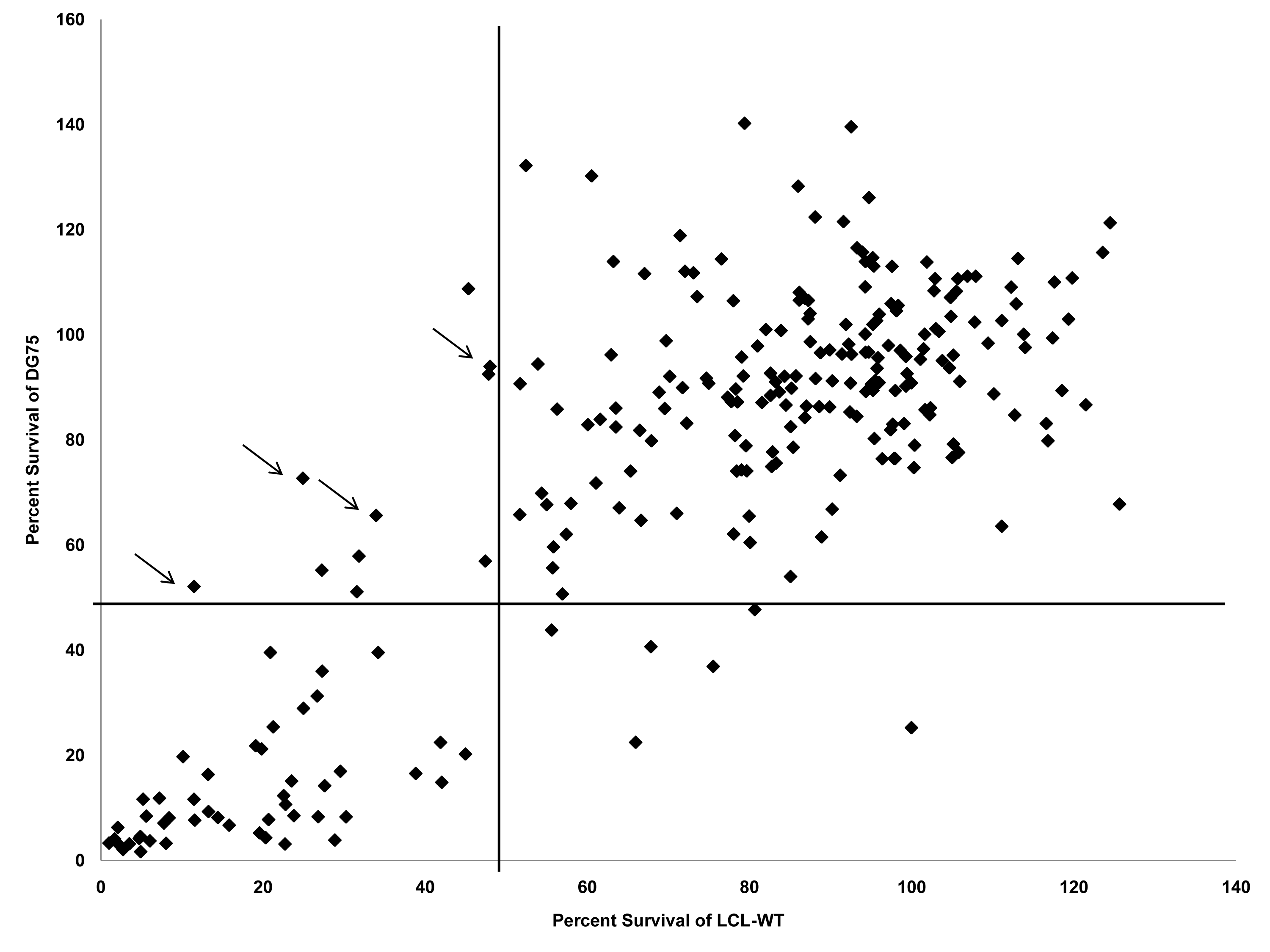

Supplement: Figure S1 — Initial screening of the Chemical Validation Library. LCL- WT and DG75 cells were treated with each one of 254 kinase inhibitors (1 µM) of the Chemical Validation Library for 4 days. The results are the means from three independent experiments. Compounds that inhibited the viability of LCL-WT by at least 50% but did not reduce the viability of DG75 cells by more than 50% were tested further for their effect against an additional EBV-transformed B cell line (LCL-FLAG-LMP1) and PBMCs. The four inhibitors (PP2, compound 5, CI-1040 and PD 198306) that were found to compromise the viability of EBV-positive cells preferentially and analyzed in the present study are indicated by arrows. The rest of the inhibitors of the upper left quadrant were not analyzed further because they did not exhibit similar results, when tested against LCL-FLAG-LMP1 and PBMCs. (TIF) [file pone.0095688.s001.tif]
